# Effect of Fatty Acid Amide Hydrolase Inhibitor URB597 on Orofacial Pain Perception in Rats

**DOI:** 10.3390/ijms23094665

**Published:** 2022-04-23

**Authors:** Marek Zubrzycki, Maria Zubrzycka, Grzegorz Wysiadecki, Janusz Szemraj, Hanna Jerczynska, Mariusz Stasiolek

**Affiliations:** 1 Department of Cardiac Surgery and Transplantology, The Cardinal Stefan Wyszynski Institute of Cardiology, Alpejska 42, 04-628 Warsaw, Poland; 2 Department of Clinical Physiology, Faculty of Medicine, Medical University of Lodz, Mazowiecka 6/8, 92-215 Lodz, Poland; maria.pawelska-zubrzycka@umed.lodz.pl; 3 Department of Normal and Clinical Anatomy, Chair of Anatomy and Histology, Medical University of Lodz, Żeligowskiego 7/9, 90-752 Lodz, Poland; grzegorz.wysiadecki@umed.lodz.pl; 4 Department of Medical Biochemistry, Medical University of Lodz, Mazowiecka 6/8, 92-215 Lodz, Poland; janusz.szemraj@umed.lodz.pl; 5 Central Scientific Laboratory (CoreLab), Medical University of Lodz, Mazowiecka 6/8, 92-215 Lodz, Poland; hanna.jerczynska@umed.lodz.pl; 6 Department of Neurology, Medical University of Lodz, Kopcinskiego 22, 90-153 Lodz, Poland; mariusz.stasiolek@umed.lodz.pl

**Keywords:** endocannabinoids, URB597, CBR, neuropeptides, orofacial pain

## Abstract

Endocannabinoids act as analgesic agents in a number of headache models. However, their effectiveness varies with the route of administration and the type of pain. In this study, we assessed the role of the fatty acid amide hydrolase inhibitor URB597 in an animal model of orofacial pain based on tooth pulp stimulation. More specifically, we assessed the effects of intracerbroventricular (i.c.v.) and intraperitoneal (i.p.) administration of URB597 on the amplitude of evoked tongue jerks (ETJ) in rats. The levels of the investigated mediators anandamide (AEA), 2-arachidonyl glycerol (2-AG), Substance P (SP), calcitonin-gene-related peptide (CGRP), endomorphin-2 (EM-2) and fatty acid amide hydrolase (FAAH) inhibitor by URB597 and receptors cannabinoid type-1 receptors (CB1R), cannabinoid type-2 receptors (CB2R) and µ-opioid receptors (MOR) were determined in the mesencephalon, thalamus and hypothalamus tissues. We have shown that increasing endocannabinoid AEA levels by both central and peripheral inhibition of FAAH inhibitor by URB597 has an antinociceptive effect on the trigemino-hypoglossal reflex mediated by CB1R and influences the activation of the brain areas studied. On the other hand, URB597 had no effect on the concentration of 2-AG in the examined brain structures and caused a significant decrease in CB2R mRNA expression in the hypothalamus only. Tooth pulp stimulation caused in a significant increase in SP, CGRP and EM-2 gene expression in the midbrain, thalamus and hypothalamus. In contrast, URB597 administered peripherally one hour before stimulation decreased the mRNA level of these endogenous neuropeptides in comparison with the control and stimulation in all examined brain structures. Our results show that centrally and peripherally administered URB597 is effective at preventing orofacial pain by inhibiting AEA catabolism and reducing the level of CGRP, SP and EM-2 gene expression and that AEA and 2-AG have different species and model-specific regulatory mechanisms. The data presented in this study may represent a new promising therapeutic target in the treatment of orofacial pain.

## 1. Introduction

Orofacial pain is the pain caused by damage to the branches of the trigeminal nerve or neurogenic inflammation that spreads to the orofacial area [1,2]. It accounts for approximately 10% to 26% of the adult population; in 7–11% of this population, it is classified as chronic and constitutes a serious medical and social problem [3,4]. Although the exact etiopathogenesis of most orofacial pain complaints has not been fully elucidated, recent findings suggest that the endoopioid (EOS) and endocannabinoid (ECS) systems have similar pharmacological characteristics, are frequently co-localized and exert a strong analgesic effect mainly through activation of the µ-opioid (MOR) and cannabinoid (CBR) receptors [5,6,7,8].

**Scheme 1:**
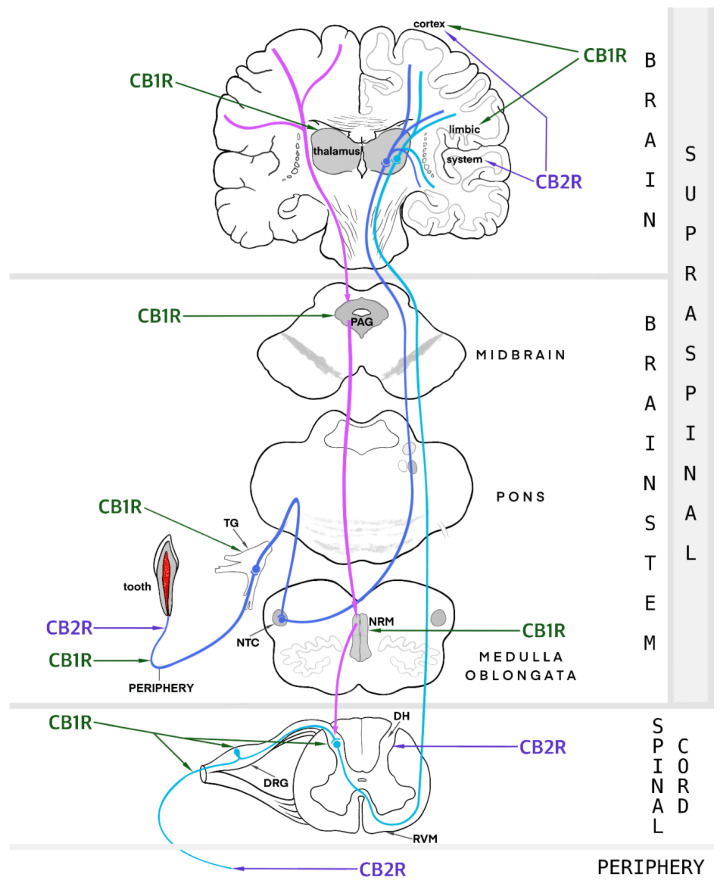
Role of the endocannabinoid system in the control of pain at the peripheral, spinal and supraspinal levels (explanations in the text). Light blue line shows the spinothalamic tract. Dark blue line indicates transmission of pain through trigeminal pathway. Violet line marks descending pain pathway. PAG—periaqueductal central gray; RVM—rostral ventromedial medulla; DRG—dorsal root ganglia; NTC—nucleus trigeminalis caudalis; DH—spinal cord dorsal horn; TG—trigeminal ganglion; CB1R—cannabinoid type-1 receptors; CB2R-cannabinoid type-1 receptors.

The cannabinoid (CB) receptors play a key role in peripheral, spinal and supraspinal nociception, including the ascending and descending pain pathways [9] (Scheme 1).

At the periphery, cannabinoid type-1 receptors (CB1R) located in nociceptive terminals inhibit nociceptive transmission, whereas cannabinoid type-2 receptors (CB2R) ones reduce the release of pronociceptive agents [10,11]. Moreover, CB1Rs are expressed in the dorsal root ganglia (DRG) and in the spinal cord dorsal horn (DH), where they inhibit neurotransmitter release and pain transmission [8,12]. At the supraspinal level, CB1Rs inhibit ascending nociceptive transmission and activate the inhibitory descending pathway of pain through the inhibition of gamma aminobutyric acid (GABA) release in the periaqueductal central gray (PAG) and rostral ventromedial medulla (RVM) [13,14].

Pain information from the orofacial area reaches the trigeminal ganglion (TG) and the nucleus trigeminalis caudalis (NTC) through the primary afferents of the trigeminal nerve [15,16,17,18]. The activated NTC transmits pain signals to higher brain centers, including the mesencephalon, thalamus, hypothalamus and the somatosensory cortex. Increased activation of neurons in the TG trigeminal ganglia may lead to reduced levels of endocannabinoid anandamide (AEA), which may in turn lead to an increase in the calcitonin gene peptide (CGRP) and Substance P (SP) release [16,19,20,21]. Low levels of the two main endocannabinoids, AEA and 2-arachidonoylglycerol (2-AG), have been correlated with headaches and provide clinical evidence for the theory of endocannabinoid deficiency, which underlies the pathophysiology of orofacial pain [22,23,24,25,26]. It was previously observed that endocannabinoids inhibit, through a CB1R-dependent retrograde mechanism, the release of neuropeptides and neurotransmitters controlling nociceptive inputs [16,19,21] and that their levels are high in the regions known to be involved in transmission and modulation of pain signals including PAG, RVM and NTC [27,28,29]. Inactivation of endocannabinoid signaling occurs through enzymatic hydrolysis mediated by two major enzymes, hydrolases fatty acid amide hydrolase (FAAH) and the monoacylglycerol lipase (MAGL), responsible for the degradation of AEA and 2-AG, respectively [30]. The action of FAAH inhibitors is based on extending the biological effects of AEA, and also endocannabinoid-like congeners, by increasing their levels in the peripheral (PNS) and central nervous system (CNS). Recent pharmacological studies have shown that inhibition of FAAH increases ECS activity and causes analgesia and reduces inflammation in animal models of migraine [21,31,32], but little is known about its effects in orofacial pain. Recently collected data suggest a strong contribution of peripheral mechanisms in pain suppression [22,31,33]. One way to explore the therapeutic potential of peripheral-endocannabinoid-mediated analgesia is to use CNS-penetrant inhibitors of the anandamide-degrading enzyme FAAH [21,31,34]. Since the currently recommended pharmacological treatments for orofacial pain are of low effectiveness and often cause many troublesome side effects, we decided to investigate the effect of centrally and peripherally administered FAAH inhibitor URB597 on the mRNA expression levels of genes encoding selected neuropeptides and on AEA, 2-AG levels and their receptors in the brain structures important for the generation and propagation of pain signals specific to orofacial pain. A better understanding of the genetic disorders associated with orofacial pain can improve prognosis and prognostication and help in the development of modern, effective treatments for this pain.

## 2. Results

### 2.1. Influence of AEA, 2-AG, EM-2 and URB597 Perfusion of Cerebral Ventricles on the Amplitude of the Trigemino-Hypoglossal Reflex in Rats

**Figure 1:**
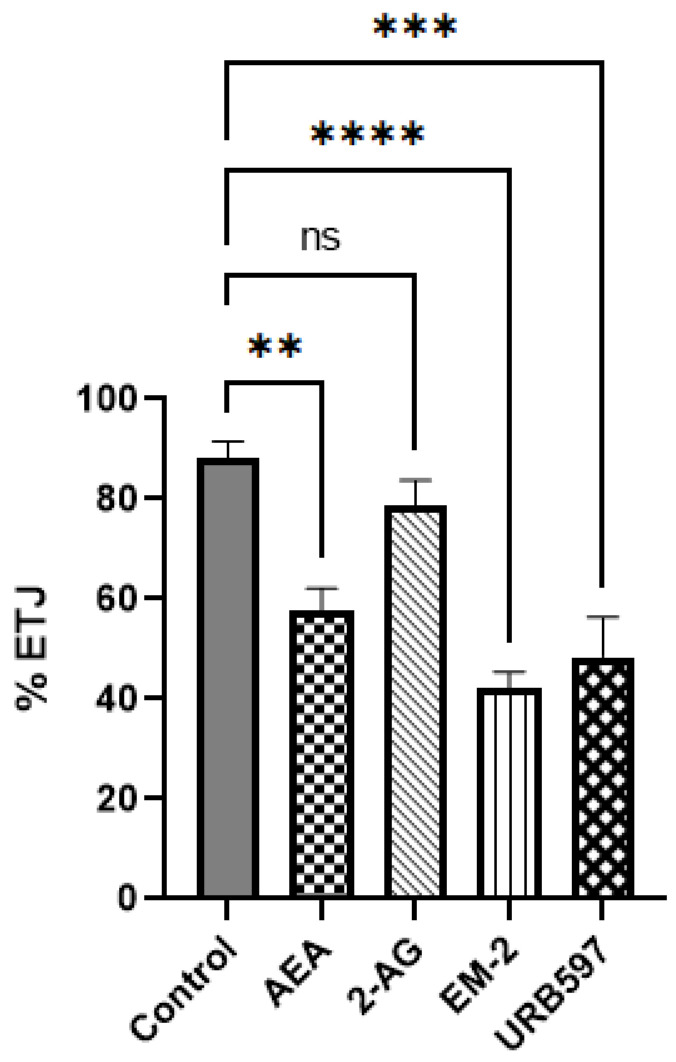
Effects of perfusion of cerebral ventricles with aCSF and with AEA, 2-AG, EM-2 and URB597 in rats on ETJ induced by tooth pulp stimulation. The data represent mean ± SEM of six rats per group. The highest amplitude of ETJ after i.c.v. perfusion with aCSF in control group was considered a 100% response. Statistical analyses were performed using one-way ANOVA and a post hoc multiple comparison Tukey test. Asterisks denote significance level (** *p* < 0.01, *** *p* < 0.001, **** *p* < 0.0001, ns—not statistically significant).

Perfusion of cerebral ventricles with AEA caused a significant reduction (*p* = 0.0027) in the amplitude of ETJ, as compared to control perfusion with aCSF. Similarly, perfusion with EM-2 and URB597 also had analgesic activity and caused a decrement in the ETJ amplitude (*p* ≤ 0.0001 and *p* = 0.0001, respectively). The perfusion with 2-AG did not show antinociceptive activity compared to the aCSF (*p* = 0.6982). The impact of the signaling molecules on the ETJ is presented in Figure 1.

### 2.2. Influence of Tooth Pulp Stimulation and Peripheral URB597 Administration on MRNA Levels of Neurotransmitters (SP, CGRP and EM-2) in the Brain Structures of Rats

Next, the changes in the transcription of selected signaling molecules were evaluated in the presence of pain stimuli and the FAAH inhibitor, URB-597, which reduces orofacial pain.

**Figure 2:**
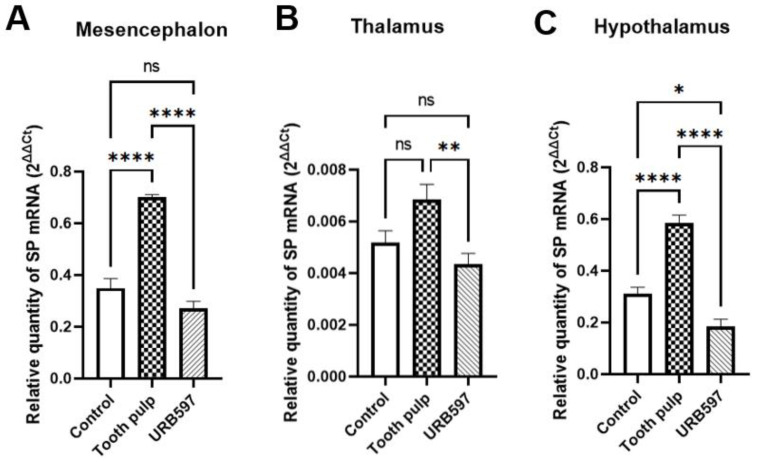
Quantitative RT-PCR analysis of SP mRNA expression in (**A**) mesencephalon, (**B**) thalamus and (**C**) hypothalamus induced by tooth pulp and URB597 treatment. The data represent mean ± SEM of 6 rats per group. Statistical significance was assessed using one-way ANOVA and a post hoc multiple comparison Tukey test. Asterisks denote significance level (* *p* < 0.05, ** *p* < 0.01, **** *p* < 0.0001, ns—not statistically significant).

SP mRNA expression was similar in the mesencephalon and hypothalamus, whereas lower expression levels were observed in thalamus. There were significant differences in SP expression levels in response to nociceptive stimulation and the URB597 treatment group in mesencephalon (F (2, 15) = 69.31, *p* < 0.0001), thalamus (F (2, 15) = 6.341, *p* = 0.0101) and hypothalamus (F (2, 15) = 51.26, *p* < 0.0001) (Figure 2). Tooth pulp stimulation caused a significant increase in SP mRNA expression compared to the control in the mesencephalon (*p* < 0.0001) and hypothalamus (*p* < 0.0001), and a similar trend was observed in the thalamus, but it was not statistically significant (*p* = 0.0819). In all three brain structures, the mesencephalon, thalamus and hypothalamus, treatment with URB597 caused downregulation of SP mRNA expression compared to a group with nociceptive stimulation (*p* < 0.0001, *p* = 0.0086 and *p* < 0.0001, respectively).

**Figure 3:**
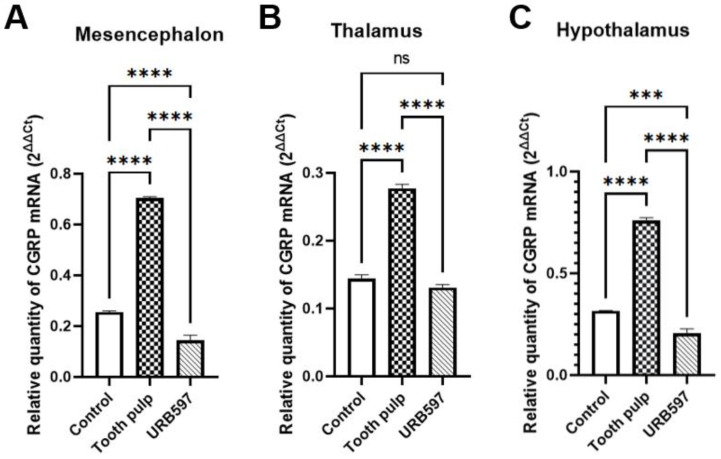
Quantitative RT-PCR analysis of CGRP mRNA expression in (**A**) mesencephalon, (**B**) thalamus and (**C**) hypothalamus induced by tooth pulp and URB597 treatment. The data represent mean ± SEM of 6 rats per group. Statistical significance was assessed using one-way ANOVA and a post hoc multiple comparison Tukey test. Asterisks denote significance level (*** *p* < 0.001, **** *p* < 0.0001, ns—not statistically significant).

In all three analyzed brain structures, nociceptive stimulation and antinociceptive treatment caused significant deregulation of CGRP mRNA expression (mesencephalon-F (2, 15) = 563.6, *p* < 0.0001, thalamus-F (2, 15) = 253.1, *p* < 0.0001) and hypothalamus-F (2, 15) = 360.4, *p* < 0.0001) (Figure 3). In the tooth pulp stimulation group, higher expression of CGRP mRNA was observed in all structures compared to the controls. URB597 treatment caused downregulation of CGRP mRNA expression compared to controls, but in the thalamus, this change was not statistically significant (*p* = 0.1736). 

**Figure 4:**
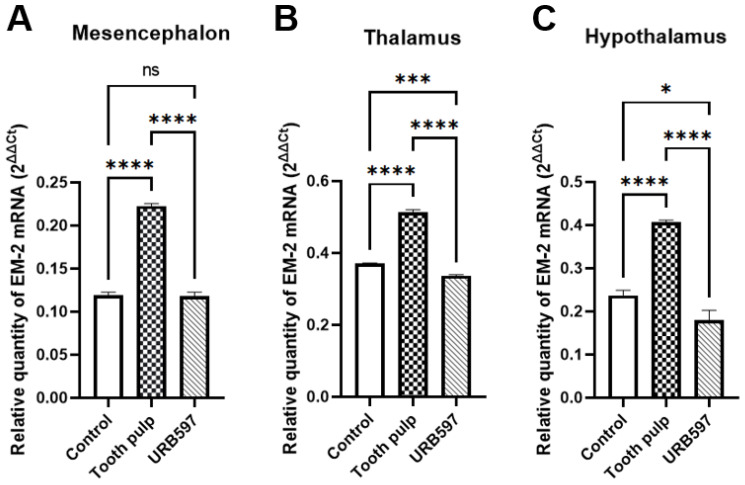
Quantitative RT-PCR analysis of EM-2 mRNA expression in (**A**) mesencephalon, (**B**) thalamus and (**C**) hypothalamus induced by tooth pulp and URB597 treatment. The data represent mean ± SEM of 6 rats per group. Statistical significance was assessed using one-way ANOVA and a post hoc multiple comparison Tukey test. Asterisks denote significance level (* *p* < 0.05, *** *p* < 0.001, **** *p* < 0.0001, ns—not statistically significant).

Expression of EM-2 mRNA was also significantly changed in response to nociceptive stimulation and URB-597 treatment in the brain structures (mesencephalon-F (2, 15) = 257.0, *p* < 0.0001; thalamus-F (2, 15) = 449.3, *p* < 0.0001; hypothalamus-F (2, 15) = 62.83, *p* < 0.0001) (Figure 4). In all structures, tooth pulp stimulation caused higher expression of EM-2 mRNA. Similarly, the antinociceptive treatment caused downregulation of EM-2 in the thalamus (*p* = 0.0002) and hypothalamus (*p* = 0.0406). There was no change in EM-2 mRNA expression in response to URB597 treatment in the mesencephalon (*p* = 0.9571). The detailed results of tooth pulp stimulation and URB597 treatment on the levels of signaling mediators are provided in Supplementary Table S1.

### 2.3. Influence of Tooth Pulp Stimulation and Peripheral URB597 Administration on MRNA Levels of CB1R, CB2R and MOR in the Brain Structures of Rats

**Figure 5:**
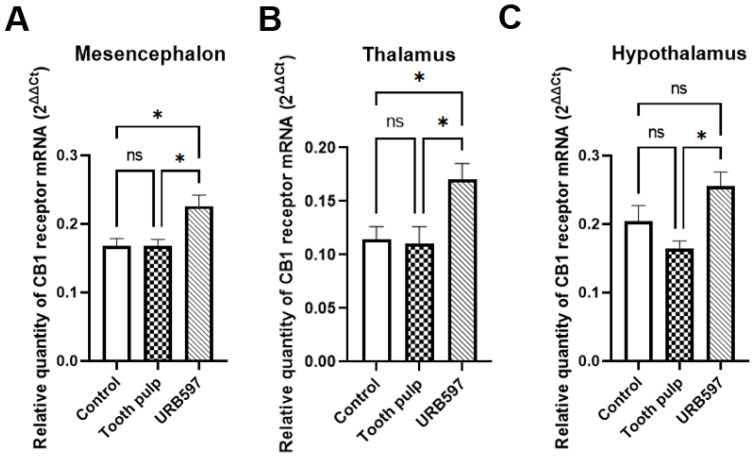
Quantitative RT-PCR analysis of CB1 receptor mRNA expression in (**A**) mesencephalon, (**B**) thalamus and (**C**) hypothalamus induced by tooth pulp and URB597 treatment. The data represent mean ± SEM of 6 rats per group. Statistical significance was assessed using one-way ANOVA and a post hoc multiple comparison Tukey test. Asterisks denote significance level (* *p* < 0.05, ns—not statistically significant).

According to ANOVA results, changes in CB1R expression in brain structures were observed, which were dependent on pain stimuli and antinociceptive treatment (mesencephalon-F (2, 15) = 7.217, *p* = 0.0064; thalamus-F (2, 15) = 5.417, *p* = 0.0169; hypothalamus F (2, 15) = 5.821, *p* = 0.0135) (Figure 5). In the mesencephalon, CB1R expression was upregulated in response to URB597 peripheral treatment compared to the control (*p* = 0.0129) and tooth pulp stimulation (*p* = 0.0129). Similar results were observed in the thalamus compared to the control and tooth pulp stimulation groups (*p* = 0.0382 and *p* = 0.0253, respectively). Upregulation of CB1R was also noted in the hypothalamus compared to nociceptive stimulation (*p* = 0.0382). 

**Figure 6:**
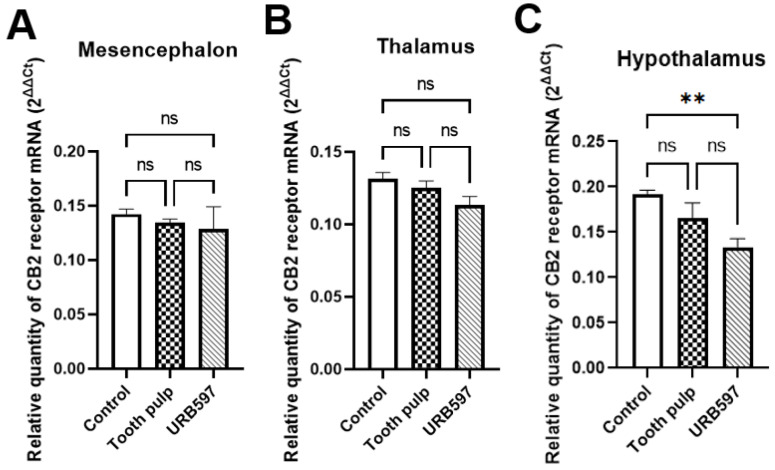
Quantitative RT-PCR analysis of CB2 receptor mRNA expression in (**A**) mesencephalon, (**B**) thalamus and (**C**) hypothalamus induced by tooth pulp and URB597 treatment. The data represent mean ± SEM of 6 rats per group. Statistical significance was assessed using one-way ANOVA and a post hoc multiple comparison Tukey test. Asterisks denote significance level (** *p* < 0.01, ns—not statistically significant).

Both nociceptive stimulation and antinociceptive treatment caused significant changes in CB2R mRNA expression only in the hypothalamus (mesencephalon F (2, 15) = 0.3265, *p* = 0.7265; thalamus (2, 15) = 3.261, *p* = 0.0667; hypothalamus F (2, 15) = 6.442, *p* = 0.0096) (Figure 6). Downregulation of CB2R expression in response to URB597 treatment was observed compared to the control (*p* = 0.0072). 

**Figure 7:**
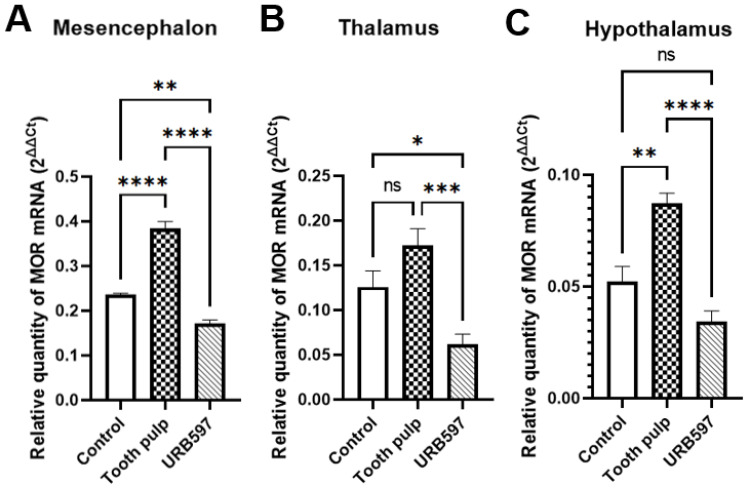
Quantitative RT-PCR analysis of MOR mRNA expression in (**A**) mesencephalon, (**B**) thalamus and (**C**) hypothalamus induced by tooth pulp and URB597 treatment. The data represent mean ± SEM of 6 rats per group. Statistical significance was assessed using one-way ANOVA and a post hoc multiple comparison Tukey test. Asterisks denote significance level (* *p* < 0.05, ** *p* < 0.01, *** *p* < 0.001, **** *p* < 0.0001, ns—not statistically significant).

Significant changes in MOR mRNA expression were found in response to tooth pulp stimulation and URB597 treatment in all analyzed brain structures (mesencephalon F (2, 15) = 114.8, *p* < 0.0001; thalamus F (2, 15) = 11.39, *p* = 0.0010; hypothalamus F (2, 15) = 24.48, *p* < 0.0001) (Figure 7). Nociceptive stimulation caused upregulation of MOR mRNA expression in all structures compared to the controls, but in the thalamus, this difference was not statistically significant (*p* = 0.1483). On the other hand, antinociceptive treatment downregulated MOR compared to the controls, and this decrease did not meet statistical significance in the hypothalamus (*p* = 0.0846). The detailed results of tooth pulp stimulation and peripheral URB597 administration on mRNA levels of CB1R, CB2R and MOR in the brain structures of rats are provided in Supplementary Table S2.

### 2.4. Influence of Tooth Pulp Stimulation and Peripheral URB597 Administration on AEA and 2-AG Concentration in the Brain Structures of Rats

**Figure 8:**
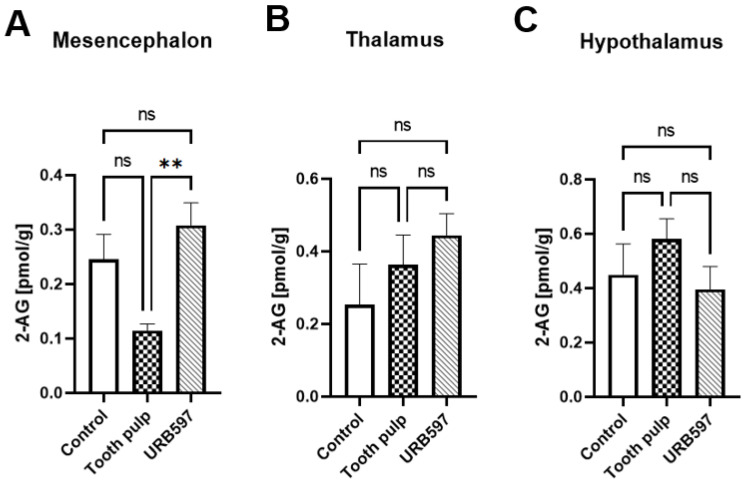
2-AG concentration in rats in controls and after tooth pulp and URB597 treatment (**A**–**C**). The data represent mean ± SEM of 6 rats per group. Statistical significance was assessed using one-way ANOVA and a post hoc multiple comparison Tukey test. Asterisks denote significance level (** *p* < 0.01, ns—not statistically significant).

**Figure 9:**
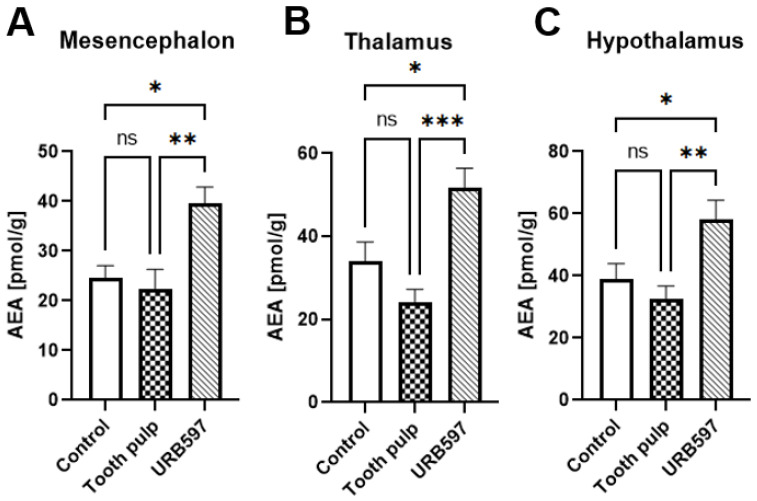
AEA concentration in rats in controls and after tooth pulp and URB597 treatment (**A**–**C**). The data represent mean ± SEM of 6 rats per group. Statistical significance was assessed using one-way ANOVA and a post hoc multiple comparison Tukey test. Asterisks denote significance level (* *p* < 0.05, ** *p* < 0.01, *** *p* < 0.001, ns—not statistically significant).

In the next step, we investigated whether nociceptive stimulation or antinociceptive treatment with URB597 would impact 2-AG concentration in brain structures (Figure 8 and Figure 9). We found that 2-AG concentration did not differ between groups in the thalamus (F (2, 15) = 1.221, *p* = 0.3226) or hypothalamus (F (2, 15) = 1.090, *p* = 0.3615). In the mesencephalon, 2-AG concentration dropped in the tooth pulp stimulation group (F (2, 15) = 7.324, *p* = 0.0060), but this difference was statistically significant in relation to URB597 treatment (*p* = 0.0051) and was just above the significance level in comparison to the control (*p* = 0.0574). 

We found significant differences in AEA concentration in all investigated brain structures among the groups (mesencephalon-F (2, 15) = 7.917, *p* = 0.0045; thalamus-F (2, 15) = 10.85, *p* = 0.0012; and hypothalamus F (2, 15) = 6.590, *p* = 0.0088) (Figure 9). There were no significant differences between the tooth pulp stimulation group and controls in any of the structures. On the other hand, peripheral URB597 treatment significantly increased AEA concentration in the mesencephalon (*p* = 0.0062), thalamus (*p* = 0.0010) and hypothalamus (*p* = 0.0087). Similar results were obtained when the AEA concentration was normalized per milligram of total protein concentration in the weighed tissue (Supplementary Figures S1 and S2). The detailed results of effects of tooth pulp stimulation and peripheral URB597 administration on AEA and 2-AG concentration in the brain structures of rats are provided in Supplementary Table S3.

## 3. Discussion

The ECS plays an important role in the transmission of trigeminal pain by releasing various neurotransmitters from nerve endings that induce peripheral and central sensitization [20,21]. Previous studies have shown that FAAH inhibitors that act by increasing endocannabinoid levels and extending their duration of action without eliciting the psychoactive effects related to direct CB1 receptor activation may offer a promising treatment option for headaches [21,22,25,31,34]. This strategy of increasing endocannabinoids by inhibiting their degrading enzymes has the benefit of activating cannabinoid receptors at the sites of nociceptive pathways with high endocannabinoid turnover rather than global activation of CB1 receptors, which can result in side effects [22,29,35]. In order to learn about the involvement of the ECS in the pathophysiology of orofacial pain, we decided to investigate the influence of central and peripheral administration of the FAAH inhibitor URB597 on the trigemino-hypoglossal reflex induced by tooth pulp stimulation. 

We have shown that cerebral ventricles AEA, URB597 and endomorphin-2 (EM-2) perfusion lowered the amplitude of the ETJ reflex because the cell receptors of the neurons that conduct these impulses in the trigemino-hypoglossal reflex arc are probably saturated with endogenous cannabinoids and opioids. It can also be assumed that these compounds, when given intracerebroventricularly (i.c.v.), quickly penetrated the ventricular lining and exerted an effect on the adjacent structures, producing an analgesic effect, as the elements of the trigemino-hypoglossal reflex arc are located near the cerebral ventricles. In contrast, 2-AG showed lower efficacy and had no significant effect on the ETJ amplitude in this experiment. A plausible explanation for this negative result is that a more robust noxious stimulus may be needed for 2-AG to be mobilized. This finding is not clear and requires further investigation. 

Since the ECS may control the release of mediators involved in headache [21,24], as a further test in this study we examined the effect of peripherally administered FAAH inhibitor URB597 and tooth pulp stimulation on mRNA levels of selected mediators, including endocannabinoids and their receptors, in the brain structures involved in the processing of pain signals from the orofacial area in rats. We observed that tooth pulp stimulation caused a significant increase in the expression of the SP, CGRP and EM-2 genes in the midbrain, thalamus and hypothalamus. On the other hand, peripheral administration of URB597 one hour before stimulation decreased the mRNA level of these endogenous neuropeptides compared to the control and stimulated all examined brain structures. This finding suggests that URB597 increases the AEA levels in the brain, which in turn leads to decreased activation of the neuropeptidergic pathways.

**Scheme 2:**
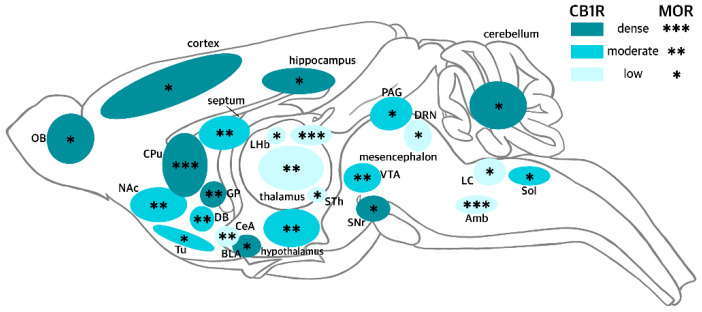
Schematic representation of CB1R and MOR distribution in the rat brain. Figure taken from Befort [36] under the terms of the Creative Commons Attribution License (CC BY). Authors’ own modification. The distribution of CB1Rs and MOR is indicated by number of stars with a low (*), moderate (**), and dense (***) expression. Amb—ambiguous nucleus; BLA—basolateral nucleus of the amygdala; CeA—central amygdala; CPu—caudate putamen; DB—diagonal band; DRN—dorsal raphe nucleus; GP–globus pallidus; LC, locus coeruleus; LHb, lateral habenular nucleus; mHb, medial habenular nucleus; NAc—nucleus accumbens; OB–olfactory bulb; PAG—periaqueductal central gray; SNr—substantia nigra pars reticulate; Sol—nucleus of the solitary tract; STh—subthalamic nucleus (ventral thalamus); Tu—olfactory tubercle; VTA—ventral tegmental area.

The biological effects of endocannabinoids and opioids in humans and animals are mainly related to the distribution of their specific receptors in the CNS [7,36,37,38] (Scheme 2).

Therefore, as another test of this hypothesis, we investigated the effect of tooth pulp stimulation and peripheral administration of URB597 on the expression of CB1R, CB2R and MOR in the midbrain, thalamus and hypothalamus of rats. We observed upregulation of CB1R in each of the brain regions after administration of URB597, while in the hypothalamus, we additionally noted downregulation of CB1R after stimulation with tooth pulp. CB1 receptors were likely activated by the increased levels of AEA in the midbrain, thalamus and hypothalamus produced by administering URB597 before stimulation. These observations suggest that the analgesic effect of URB597 requires the CB1R, confirming the role of this receptor in the response to orofacial pain. In another study, it was found that CB1 receptors expressed in peripheral trigeminal nerve endings can contribute to antinociception by reducing the probability of spike generation and decreased neurotransmitter release [21,39]. 

On the other hand, stimulation of the tooth pulp and URB597 caused changes in the expression of CB2R mRNA only in the hypothalamus, where downregulation of CB2R expression in response to URB597 administration compared to the control was observed. This may be because the signaling of CB1 and CB2 receptors is pleiotropic and it is difficult to activate these receptors directly without affecting other receptors, such as TRPV1 [40,41,42], PPARy [43,44], GABA A [45,46,47], Adenosine A3 [48] and GPR55 [43,49], by binding to endocannabinoids, which can exert physiological effects. Moreover, CB receptors form both homo- and heterodimers and undergo allosteric modulation, which correlates with the receptor functionality and anatomical localization [50,51,52].

Since endocannabinoids interact with opioids and vice versa to enhance the analgesic effect [6,10,50], in the present study we also analyzed the effect of tooth pulp stimulation and URB597 administration on MOR mRNA expression in the midbrain, thalamus and hypothalamus. We observed that tooth pulp stimulation caused upregulation of MOR mRNA expression, and URB597 administration lowered its expression in all brain structures. These observations suggest that the stimulation of the tooth pulp may lead to increased axonal transport of MOR receptors to the sensory fiber terminals through mechanisms that directly or indirectly increase in their synthesis in the brain, as well as confirm the ability of URB597 to modulate the pronociceptive effects.

At the next stage, we investigated whether nociceptive stimulation or i.p. administration of URB597 would impact AEA and 2-AG concentration in the mesencephalon, thalamus and hypothalamus. We observed that tooth pulp stimulation had no effect on the mobilization of AEA and 2-AG compared to controls in any of the brain structures, while URB597 caused a significant increase in AEA concentration in the midbrain, thalamus and hypothalamus, i.e., in the regions where this lipid messenger is produced de novo on demand [53]. These findings are consistent with other reports that URB597 increases brain AEA levels in rats and mice [21,35,54,55,56,57]. 

On the other hand, URB597 had no significant effect on the concentration of 2-AG in the examined brain structures. Inhibition of FAAH activity by URB597 resulted in an increase in AEA and CB1 receptor levels, as well as a decrease in CB2 receptor expression. Interestingly, 2-AG was less effective in our experiments despite the fact that it is present in higher concentrations in the brain than AEA and can induce stimulation equally through CB1 and CB2 receptors [58]. It may be due to lower levels of 2-AG access to the presynaptic CB1 receptors, as well as to the fact that the 2-AG-degrading enzyme is closer to the target of the CB1R than AEA. It should also be taken into account that the production and synthesis of AEA and 2-AG can occur independently in the cell and can often cause contradictory effects [59,60,61], or that the FAAH/MAGL activity profile is different in the TG associated with orofacial pain and the DRG involved in transmission of this pain. Additionally, the measurements of headache sensitivity after DAGL inhibition suggest that reduced 2-AG signaling in the cortex and PAG, but not in the NTC or TG, drives the initiation of headache [26]. Our research shows that trigeminal neurons do not always respond in the same way as spinal neurons with respect to somatosensory modulation, as was also confirmed in the studies by Akerman et al. [62] and Levine et al. [26]. Furthermore, our findings are consistent with the theory that AEA and 2-AG can independently regulate pain perception as they have different experimental model-specific and animal-species-specific regulatory mechanisms [63,64]. This may further complicate the interpretation of our results and necessitates further research into its function in orofacial pain.

There is evidence to suggest that dysregulation of endocannabinoid signaling may contribute to the etiology and pathophysiology of migraine headache [21,62,65,66,67,68]. In an animal model of nitroglycerin-induced migraine, systemic administration of the FAAH inhibitor, URB597 attenuated nitroglycerin-induced mechanical hyperalgesia in mice and rats, indicating elevation of the AEA level by the FAAH inhibitors and a reduction in migraine pain [21,66]. The action of the FAAH inhibitor was antagonized by a CB1 receptor antagonist rimonabant [66]. The administration of the FAAH inhibitor also reduced nitroglycerin-induced neuronal c-Fos protein expression in the brainstem trigeminal nuclei, which was blocked by a CB1 receptor antagonist [21,66,69]. Additionally, knockout of FAAH but not MAGL in mice attenuated nitroglycerin-induced mechanical hyperalgesia, indicating a crucial role of AEA in attenuation of pain [66]. In another study, the brain-impermeable FAAH inhibitor URB937 was effective at reducing acute and chronic trigeminal hyperalgesia induced by nitroglycerin, suggesting that an increase in AEA levels and activation of CB1 receptors in peripheral nerves and tissues may reduce migraine pain and produce fewer side effects as compared with those acting in the CNS [68,70].

Taken together, our results suggest that peripheral and central inhibition of FAAH by URB597 demonstrated analgesic effect in a rat model orofacial pain. These data confirm the ability of URB597 to modulate the analgesic effects induced by tooth pulp stimulation in the orofacial area innervated by the trigeminal nerve by elevating AEA and CB1R levels. Simultaneously, the transcription of several mediator genes involved in the pathophysiology of this pain was attenuated in the mesencephalon, thalamus and hypothalamus. Our results provide indirect support for the hypothesis that AEA and 2-AG in the periphery differentially suppress pain transmission initiated by tooth pulp stimulation, and the effects are likely to be dependent upon the specific endocannabinoid elevated. This peripheral FAAH inhibition may exert tight regulatory control over pain processing and may offer a more effective pain management strategy than global inhibition of FAAH that circumvents the limitations of orthosteric agonists at the cannabinoid receptors. The results support a role of AEA-mediated endocannabinoid signaling in migraine and suggest that FAAH may offer a new therapeutic option for the prevention of orofacial pain. However, clinical research in this area is still at an early stage, and some initial setbacks introduce a note of caution. Therefore, further experimental research is needed to confirm this speculation.

### Limitations

The authors acknowledge that the studies described herein have limitations. 

In the present study, we have confirmed the use of the FAAH inhibitor URB597 as a potential therapy for orofacial pain [21]. However, additional research is needed to investigate the changes in AEA, 2-AG and endocannabinoid congeners levels in the central trigeminal structures. Our observations provide evidence for the involvement of the ECS in orofacial pain, but do not provide conclusive data on the comparison of peripherally limited effects compared to a non-peripherally restricted FAAH inhibitor. For this purpose, further direct comparative studies should be carried out.

## 4. Materials and Methods

### 4.1. Animals and Anesthesia

Adult male Long–Evans rats, weighing 330–350 g and at 3–4 weeks of age, were randomly allocated to experimental groups in this study. The animals were housed at a constant temperature (22–24 °C) and maintained in sawdust-lined plastic cages under a 12 h light–dark cycle with free access to laboratory chow pellets and tap water ad libitum. The rats were anesthetized with a single i.p. injection of chloralose solution at a dose of 150 mg·kg^−1^ body weight. In all the experimental animals, the anesthesia persisted for the whole period of the experiment. For each experiment, groups of 6 animals were used. Animal sample sizes were determined based on our preliminary studies to ensure adequate statistical power. To minimize circadian influence, all experiments were performed between 8:00 a.m. and 12:00 a.m. after 7 days of acclimatization. The experimental protocol was approved by the Local Ethical Committee for Animal Experiments at the Medical University of Lodz (42/ŁB 103/2018; 42/ŁB 103-A-DLZ/2020; 43/ŁB 103-B-NZP/2020) and was in accordance with the European Communities Council Directive 2010/63/EU. All experimental procedures were performed in accordance with the current guidelines for the care of laboratory animals (including the use of the 3Rs procedures) and in accordance with the ARRIVE guidelines [71,72]. 

### 4.2. Experimental Design

Control perfusion of the cerebral ventricles was conducted with aCSF, which was prepared by according to Daniel and Lederis [73] and contained 120 mM NaCl, 4.8 mM KCl, 2.8 mM CaCl_2_, 1.2 mM KH_2_PO_4_, 1.3 mM MgSO_4_, 26 mM NaHCO_3_, 10 mM glucose, 1.0 g/L bovine serum albumin and 0.1 g/L ascorbic acid (pH = 7.5). The solution was placed in a water bath at 37 °C and constantly gassed with carbogen (a mixture of 95% O_2_ and 5% CO_2_). 

**Scheme 3:**
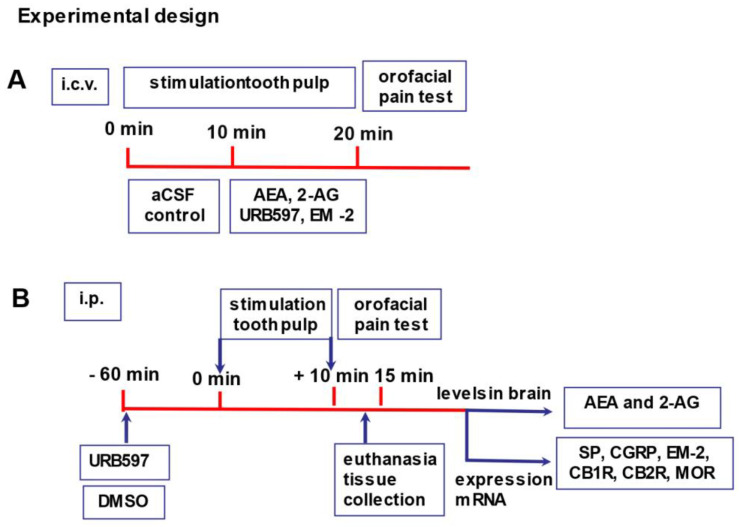
Experimental design. (**A**). Perfusion of cerebral ventricles with aCSF (control) and AEA (*n* = 6), 2-AG (*n* = 6), URB597 (*n* = 6) and EM-2 (*n* = 6) in rats for ETJ induced by tooth pulp stimulation. (**B**). One group of experimental animals which received URB597 administered intraperitoneally (i.p.) 60 min before tooth pulp stimulation was sacrificed immediately after the end of the experiment in order to determine the levels of AEA and 2-AG and mRNA expression of SP, CGRP and EM-2 mediators and CB1, CB2 and MOR receptors in rat brain (*n* = 6). The second group of experimental animals was given DMSO at 1 mL/kg i.p. (*n* = 6). At the beginning of the experimental part of this work, experiments in which ETJ was recorded without perfusion of the cerebral ventricles were carried out (*n* = 6). It was shown that the ETJ magnitude did not change during 90 min of tooth pulp stimulation while maintaining constant parameters of the noxious stimuli.

AEA, 2-AG (Tocris Bioscience, Bristol, UK), URB597 (3′-carbamoyl-biphenyl-3-yl-cyclohexylcarbamate and EM-2 (Sigma Aldrich, St. Louis, MO, USA) were administered i.c.v. at the dose of 100 nmol/mL. URB597, administered i.p., was dissolved in 100% dimethylsulfoxide (DMSO) [74] and injected at the dose of 2 mg/kg 1 h before tooth pulp stimulation (Scheme 3) [21,66]. The solution was administered at a volume of 1 mL/kg [21]. The drug doses were based on previous reports. The total number of animals used in the study was 42.

### 4.3. Perfusion of Cerebral Ventricles in Rats

The rat’s head was immobilized by introduction of ear bars into the external auditory meati and fixing the maxilla with jaw clamps in a stereotaxic instrument specially adapted for perfusion of the cerebral ventricles. Perfusion of cerebral ventricles was performed using artificial cerebrospinal fluid (aCSF), as described previously [75]. The skin of the animal’s head, anesthetized with 2% polocaine solution, was incised in the midline, and the skull bones were exposed. On the basis of modified co-ordinates given by the Paxinos and Watson stereotaxic atlas [76], the sites for drilling holes in the skull bones were determined: for the lateral ventricles, holes were drilled 9 mm anterior to the frontal interaural zero plane and 3 mm lateral to the sagittal zero plane. The system of cerebral ventricles was perfused with the investigated compounds by inserting stainless-steel cannulae into both lateral ventricles and the cerebellomedullary cistern. The container with the perfusion fluid was positioned 20 cm above the animal’s head. aCSF solution was used for perfusion. The outflow cannula inserted into the cerebellomedullary cistern was connected to a polyethylene tube ca. 100 cm long, which provided the outflow for the perfusion fluid. The flow rate at the end of the tubing in the course of perfusion was 0.5–0.7 mL/10 min.

### 4.4. Tooth Pulp Stimulation

After placing the animal’s head in a stereotaxic instrument, the tips of both lower incisors were cut off with a dental separator, and stainless-steel wire electrodes were inserted into the pulp and fixed with dental cement. The bipolar pulp stimulation was delivered 6 times per minute, with a train of four electrical impulses of 200 Hz frequency, for a duration of 3 ms per single impulse with 2 ms intervals and a 4–5 V amplitude, using a programmed stimulator. Trains of 4 impulses were delivered to the pulp at 10 s intervals using a Grass stimulator coupled with a gate generator.

### 4.5. Recording Tongue Jerks

**Scheme 4:**
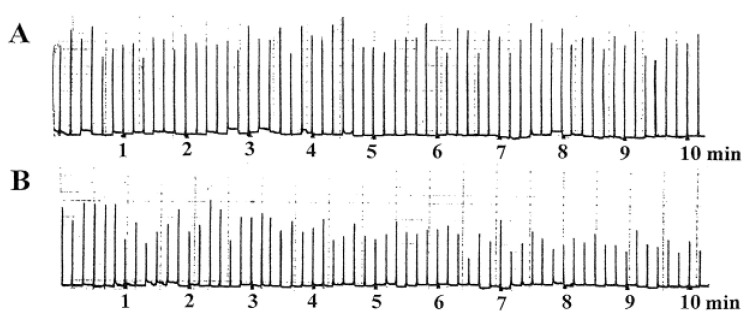
Original recordings of tongue jerks (ETJ) induced by (**A**) tooth pulp stimulation and (**B**) tooth pulp stimulation and URB597 in rat.

The tip of the animal’s tongue was attached with a silk thread to an isotonic rotating tensometric transducer. The amplitude of tongue jerks was recorded on a paper using a Line Recorder TZ-4620 (Laboratorni Pristroje, Praha, Czech). The tongue was stretched with the same force, ca. 5.8 G, throughout the experiment, and the amplification of the recorder also remained unchanged. For each animal, the amplitude of tongue jerks evoked by tooth pulp stimulation was recorded during 10 min perfusions (Scheme 4). The mean amplitude of tongue jerks evoked by tooth pulp stimulation was regarded as an indicator of magnitude of the trigemino-hypoglossal reflex.

### 4.6. Obtaining Cerebral Structures

After completion of the experiment, the rats were immediately sacrificed by decapitation. The brains were quickly removed and hardened in the freezer, and tissue bioptates from the mesencephalon, thalamus and hypothalamus were collected from each rat. The biological material for molecular studies was frozen (−70 °C) and stored for no longer than 2 weeks.

### 4.7. Preparation of Tissue Homogenate

Rat brain tissues (midbrain, thalamus and hypothalamus) were rinsed with ice-cold PBS (0.02 mol/L, pH 7.0–7.2), weighted and homogenized on ice in PBS using Tissue Raptor homogenizer (Qiagen, Hilden, Germany). The ratio of tissue weight (g) to PBS volume (mL) was 1:9. The obtained suspension was additionally frozen and thawed twice to further break down the cell membranes. The homogenates were then centrifuged for 5 min at 5000× *g* 4 °C, and obtained supernatants were aliquoted and stored at −80 °C until analysis.

### 4.8. Total Protein Concentration

The Pierce BCA Protein Assay Kit (Thermo Scientific) was used to measure total protein concentration in the homogenates. The principle of the test was based on the alkaline reduction of Cu2+ to Cu1+ by proteins and the formation of the complex bicinchoninic acid: Cu1+. Optical density was measured at 570 nm, and the standard curve for Bovine Serum Albumin (BSA) was used to determine unknown protein concentrations.

### 4.9. ELISA Test

AEA and 2-AG concentrations were measured in rat brain homogenates with commercial ELISA kits (MyBioSource, San Diego, CA, USA). The sensitivity of the tests was 0.1 ng/mL and 0.43 pg/mL for AEA and 2-AG, respectively. The protocols were performed according to the manufacturers’ instructions. A Stat-Matic Plate Washer II (Sigma-Aldrich) was used for the washing steps. The optical density was measured at 450 nm, the protein concentration of the samples was determined by interpolation from the standard curve, and intra-assay %CV was 1.42 for AEA and 5.73 for 2-AG. 

In BCA assay and ELISA, absorbance was read by VICTORTM X4 Multifunctional Microplate Reader (Perkin Elmer, Waltham, MA, USA), and the results were analyzed with WorkOut 2.5 Software. The mean concentration of protein per mL was determined with reference to a four-parameter logistic curve (4-PL).

### 4.10. RNA Isolation and Gene Expression Analysis

#### 4.10.1. Total RNA Isolation

Total RNA isolation from rat brain samples using an RNA extraction reagent, TRIZOL (Invitrogen Life Technologies, Carlsbad, CA, USA), according to the standard acid–guanidinium–phenol–chlorophorm method, was performed [77]. The absorbance of isolated RNA was measured using a spectrophotometer (Picodrop) at λ = 260 nm in order to determine total RNA concentration. The isolated RNA was stored in temperature of −70 °C.

#### 4.10.2. Quality Analysis of Isolated RNA

The quality of total RNA was checked with Agilent RNA 6000 Nano Kit (Agilent Technologies, Santa Clara, CA, USA) in accordance with the manufacturer’s recommendations. One µL of RNA 6000 Nano dye was added to a test tube containing 65 µL of Agilent RNA 6000 Nano gel matrix and then centrifuged (10 min, 13,000× *g*). The gel–fluorescent dye mixture was applied on the surface of a Nano chip placed in a workstation. Then, 5 µL of RNA Nano marker was added to selected pits. Isolated samples of RNA and RNA size markers were subject to denaturation (2 min, 70 °C), and then 1 µL of the sample was pipetted into selected pits of the Nano chip and mixed (1 min, 2400 rpm). The quality of isolated RNA was checked using 2100 Bioanalyzer (Agilent Technologies, Santa Clara, CA, USA). The level of degradation of total RNA was determined with the use of an electrophoretogram, and RIN values were recorded. Only the samples with RIN value > 7 were subject to further analysis.

#### 4.10.3. RT-PCR Reverse Transcription

An RT reaction was carried out using TaqMan^®^ RNA Reverse Transcription Kit (Applied Biosystems, Waltham, MA, USA) based on the manufacturer’s recommendations, using Rn 01511353_g1, Rn 00562002_m1, Rn 01460701_g1, Rn 04342831_g1, Rn 00668206_g1, Rn 1068079_g1 and Rn 01775763_g1 probes specifically, respectively, for rat CGPR, SP, CB1R, CB2R, MOR, EM-2 and GADPH cDNA. (Applied Biosystems). The samples were incubated (30 min, 16 °C and 30 min, 42 °C) in a thermocycler (Biometra). Reverse transcriptase was inactivated (5 min, 85 °C), and the obtained cDNA was stored at a temperature of −20 °C.

#### 4.10.4. Real-Time PCR Reaction

Real-time PCR reaction was conducted using TaqMan^®^ Universal PCR Master Mix, No UNG (Applied Biosystems, Waltham, MA, USA) according to the protocol provided by the manufacturer. The reaction mixture ratio is presented in the table. To calculate relative expression of miRNA genes, the Ct comparative method was used [78,79]. The level of rat SP, CGPR, EM-2, CB1R, CB2R, MOR and gene expression in rat brains was normalized in relation to the GADPH reference gene (Table 1).

Each target probe was amplified in a separate 96-well plate. All samples were incubated at 50 °C for 2 min and at 95 °C for 10 min and then cycled at 95 °C for 30 s, at 60 °C for 30 s and at 72 °C for 1 min; 40 cycles were performed in total. To create standard curves, 2.5, 2.0, 1.5, 1.0 and 0.5 µL of obtained cDNAs for each of the target genes was used. Fluorescence emission data were captured, and mRNA levels were quantified using the critical threshold (Ct) value. Analyses were performed with ABI Prism 7000 (SDS Software). Controls without RT and with no template cDNA were performed with each assay. Relative gene expression levels were obtained using the ∆∆Ct standard 2^−∆∆ct^ calculations and expressed as a fold change of the control sample [78,79]. Amplification-specific transcripts were further confirmed by obtaining melting curve profiles.

### 4.11. Statistical Analysis

The study was planned with the outcome as a continuous variable. Pairwise comparisons with a 1:1 ratio between groups were designed. Based on previous research, we assumed the outcome was significant when the difference between the two groups exceeded 30%, with a standard deviation of 15%. Assuming α = 0.05 and power = 0.90, we estimated that six animals per group were needed to be able to reject the null hypothesis. The PRISM 9.2 (Graph-Pad Software Inc., La Jolla, CA, USA) was used to perform statistical analysis and generate plots. The distribution of each variable was checked using the Shapiro–Wilk normality test, and any non-normally distributed variable was transformed using log function where appropriate. For nociceptive responses’ neurotransmitters, gene expression and AEA and 2-AG levels, the statistical differences between groups were assessed using one-way ANOVA followed by post hoc Tukey’s multiple comparison test. All data are presented as mean ± standard error of the mean (±SEM), and a *p*-value ≤ 0.05 was considered as statistically significant. The raw data are provided in Supplementary File S2. The data and statistical analysis comply with the experimental design and analysis recommendations in pharmacology [80].

## 5. Conclusions

In the paper, we demonstrate that URB597 significantly increased the brain AEA levels and produced CB1R-mediated antinociception and reduced the gene expression of some mediators involved in orofacial pain. Modulation of the ECS by inhibition of FAAH with URB597 administered both i.c.v. and i.p. is effective at inhibiting orofacial pain and is a promising therapeutic approach to the treatment of this pain.

## Figures and Tables

**Table 1:** PCR primer sequences used for RT-PCR analysis of mRNA.

Gene	Forward Primers	Reverse Primers
*GAPDH*	5′-GTCGCTGTTGAAGTCAGAGGAG-3′	5′-CGTGTCAGTGGTGGACCTGAC-3′
*CB1R*	5′-CTACTGGTGCTGTGTGTCATC-3′	5′-GCTGTCTTTACGGTGGAATAC-3′
*CB2R*	5′-AGGTTGCATTCCCAACAGAC-3′	5′-TTAGTTCCTCTGGGCAATGG-3′
*MOR*	5′-GTCCTTTATGCATTTCTGGATGAAAAC-3′	5′-GTCCTTTATGCATTTCTGGATGAAAAC-3′
*EM-2*	5′-GGTACCCGTTCTTCGGGAAGAAAATG-3′	5′-CCGGCATTTTCTTCCCGAAGAACGGGT-3′
*SP*	5′-GCAGTTCTTTGGATTAATGGGCA-3′	5′-TCGTAGTTCTGCATTGCGCTTC-3′
*CGRP*	5′-AGTCTCAGCTCCAAGTCATC-3′	5′-TTCCAAGGTTGACCTCAAA-3′

## Data Availability

All data generated and analyzed during this study are included in this published article (and its Supplementary Information Files).

## Supplementary Materials

The following are available online at https://www.mdpi.com/article/10.3390/ijms23094665/s1.

## Abbreviations

2-AG	2-arachidonoyl glycerol
aCSF	artificial cerebrospinal fluid
AEA	N-arachidonyl ethanol amine, Anandamide
CB	cannabinoid
CB1	cannabinoid receptor type 1
CB2	cannabinoid receptor type 2
CBR	cannabinoid receptor
CGRP	calcitonin gene related peptide
CNS	central nervous system
CSF	cerebrospinal fluid
DAGL	diacylglycerol lipase
DMSO	dimethyl sulfoxide
DRG	dorsal root ganglia
EC	endocannabinoid
ECS	endocannabinoid system
EM-2	endomorphin-2
ETJ	evoked tongue jerks
FAAH	fatty acid amide hydrolase
GABA	gamma-aminobutyric acid
MAGL	monoacylglycerol lipase
MOR	µ opioid receptor
NTC	nucleus trigeminalis caudalis
PAG	periaqueductal central gray
RVM	rostral ventromedial medulla
SP	Substance P
TG	trigeminal ganglia
TRPV1	transient receptor potential vanilloid 1
URB597	FAAH inhibitor—degrading enzyme of AEA
