# Supplementary material for: Effect of Fatty Acid Amide Hydrolase Inhibitor URB597 on Orofacial Pain Perception in Rats
Source: Int J Mol Sci. 2022 Apr 23;23(9):4665. doi: 10.3390/ijms23094665 (PMC9100922; doi:10.3390/ijms23094665)
Supplement: Supplementary file 1 [file ijms-23-04665-s001.zip › ijms-1649023-supplementary.pdf]

Supplementary Table S1. Effect of control, electrical stimulation tooth pulp, and URB597 on the levels of SP, CGRP, and EM-2 mRNAs in mesencephalon, thalamus, and hypothalamus in rats. SP, CGRP and EM-2 mRNA levels are shown as relative quantity ($2^{\Delta\Delta Ct}$) ± SEM.

Signaling mediators	Brain structure	Control	Tooth pulp	URB597	Control vs. Tooth pulp *p* - value	Control vs. URB597 *p* - value	Tooth pulp vs. URB597 *p* - value
SP	mesencephalon	0.3488 ± 0.0371	0.7007 ± 0.0101	0.2702 ± 0.0282	<0.0001	0.1415	<0.0001
	thalamus	0.0052 ± 0.0005	0.0068 ± 0.0006	0.0043 ± 0.0004	0.0819	0.4907	0.0086
	hypothalamus	0.3103 ± 0.0260	0.5847 ± 0.0310	0.1842 ± 0.0286	<0.0001	0.0182	<0.0001
CGRP	mesencephalon	0.2553 ± 0.0049	0.7040 ± 0.0056	0.1443 ± 0.0203	<0.0001	<0.0001	<0.0001
	thalamus	0.1445 ± 0.0051	0.2775 ± 0.0056	0.1308 ± 0.0044	<0.0001	0.1736	<0.0001
	hypothalamus	0.3153 ± 0.0018	0.7588 ± 0.0142	0.2043 ± 0.0226	<0.0001	0.0004	<0.0001
EM-2	mesencephalon	0.1193 ± 0.0034	0.2228 ± 0.0026	0.1178 ± 0.0049	<0.0001	0.9571	<0.0001
	thalamus	0.3710 ± 0.0015	0.5138 ± 0.0071	0.3367 ± 0.0025	<0.0001	0.0002	<0.0001
	hypothalamus	0.2372 ± 0.0117	0.4065 ± 0.0054	0.1803 ± 0.0223	<0.0001	0.0406	<0.0001

Supplementary Table S2. Effect of control, electrical stimulation tooth pulp, and URB597 on the CB1, CB2, and MOR mRNA expression levels in rat mesencephalon, thalamus, and hypothalamus. Data are presented as relative quantity ($2^{\Delta\Delta Ct}$) ± SEM.

Receptors	Brain structure	Control	Tooth pulp	URB597	Control vs Tooth pulp *p* - value	Control vs URB597 *p* - value	Tooth pulp vs URB597 *p* - value
CB1R	mesencephalon	0.1678 ± 0.0104	0.1678 ± 0.0096	0.2258 ± 0.0163	>0.9999	0.0129	0.0129
	thalamus	0.1147 ± 0.0117	0.1103 ± 0.0161	0.1703 ± 0.0149	0.9753	0.0382	0.0253
	hypothalamus	0.2045 ± 0.0224	0.1640 ± 0.0114	0.2552 ± 0.0211	0.3132	0.1751	0.0103
CB2R	mesencephalon	0.1425 ± 0.0045	0.1343 ± 0.0034	0.1285 ± 0.0206	0.8866	0.7061	0.9402
	thalamus	0.1315 ± 0.0043	0.1252 ± 0.0048	0.1132 ± 0.0062	0.6676	0.0585	0.2580
	hypothalamus	0.1922 ± 0.0043	0.1652 ± 0.0172	0.1337 ± 0.0091	0.2543	0.0072	0.1644
MOR	mesencephalon	0.2358 ± 0.0035	0.3843 ± 0.0153	0.1715 ± 0.0080	<0.0001	0.0012	<0.0001
	thalamus	0.1258 ± 0.0181	0.1722 ± 0.0188	0.0617 ± 0.0115	0.1483	0.0367	0.0007
	hypothalamus	0.0522 ± 0.0068	0.0873 ± 0.0045	0.0343 ± 0.0048	0.0010	0.0846	<0.0001

Supplementary Table S3. Effect of control, electrical stimulation tooth pulp, and URB597 on the levels of AEA and 2-AG in mesencephalon, thalamus and hypothalamus in rats. AEA and 2-AG are presented as normalized concentration [pmol/g] ± SEM.

Signaling endocannabinoids	Brain structure	Control	Tooth pulp	URB597	Control vs. Tooth pulp *p* - value	Control vs. URB597 *p* - value	Tooth pulp vs. URB597 *p* - value
AEA	mesencephalon	24.56 ± 2.52	22.34 ± 3.98	39.56 ± 3.33	0.8854	0.0159	0.0062
	thalamus	34.12 ± 4.55	24.20 ± 3.07	51.61 ± 4.81	0.2505	0.0261	0.0010
	hypothalamus	38.95 ± 4.93	32.47 ± 4.22	58.08 ± 6.22	0.6586	0.0490	0.0087
2-AG	mesencephalon	0.2450 ± 0.0466	0.1150 ± 0.0118	0.3083 ± 0.0409	0.0574	0.4547	0.0051
	thalamus	0.2533 ± 0.1117	0.3633 ± 0.0820	0.4450 ± 0.0593	0.6524	0.2938	0.7877
	hypothalamus	0.4500 ± 0.1131	0.5817 ± 0.0734	0.3967 ± 0.0823	0.5757	0.9106	0.3493

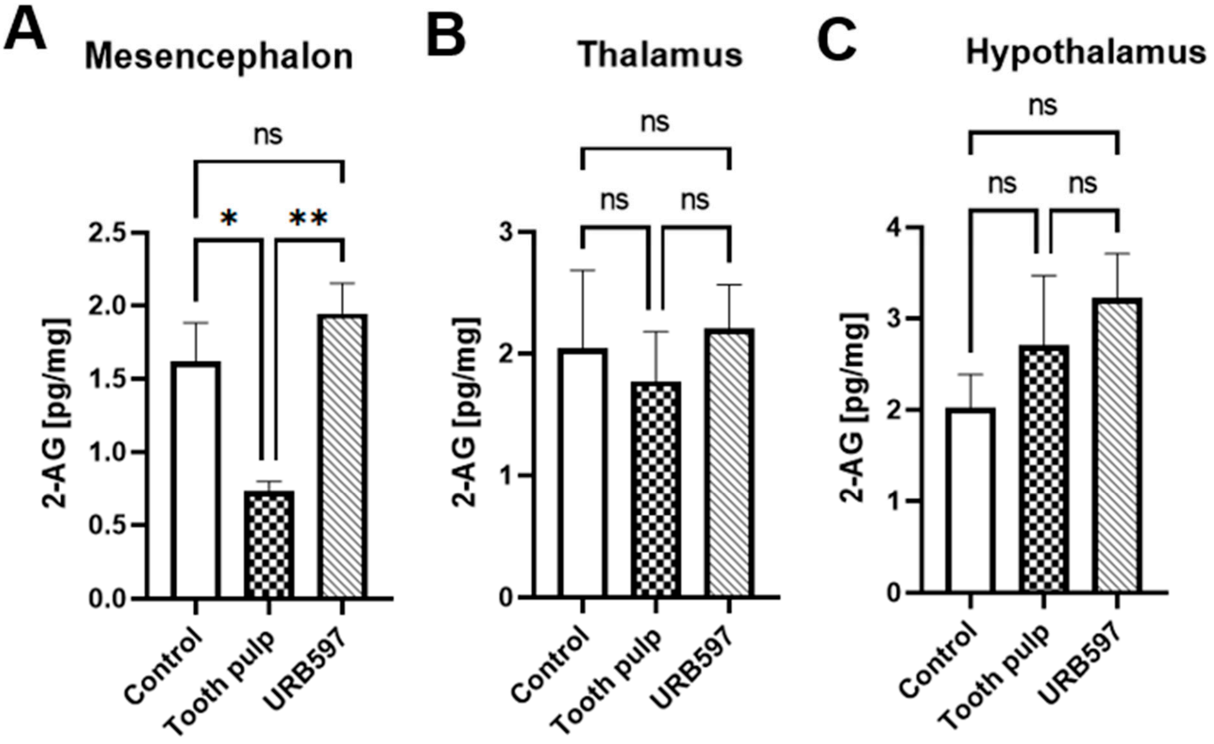

Supplementary Figure S1. 2-AG concentration normalized to milligram of total protein concentration in weighed tissue in controls and after tooth pulp and URB597 treatment. The data represent mean ± SEM of 6 rats per group. Statistical significance was assessed using one-way ANOVA and a post-hoc multiple comparison Tukey test. Asterisks denote significance level (* <0.05, **<0.01, *** <0.001, **** <0.0001, ns- not statistically significant).

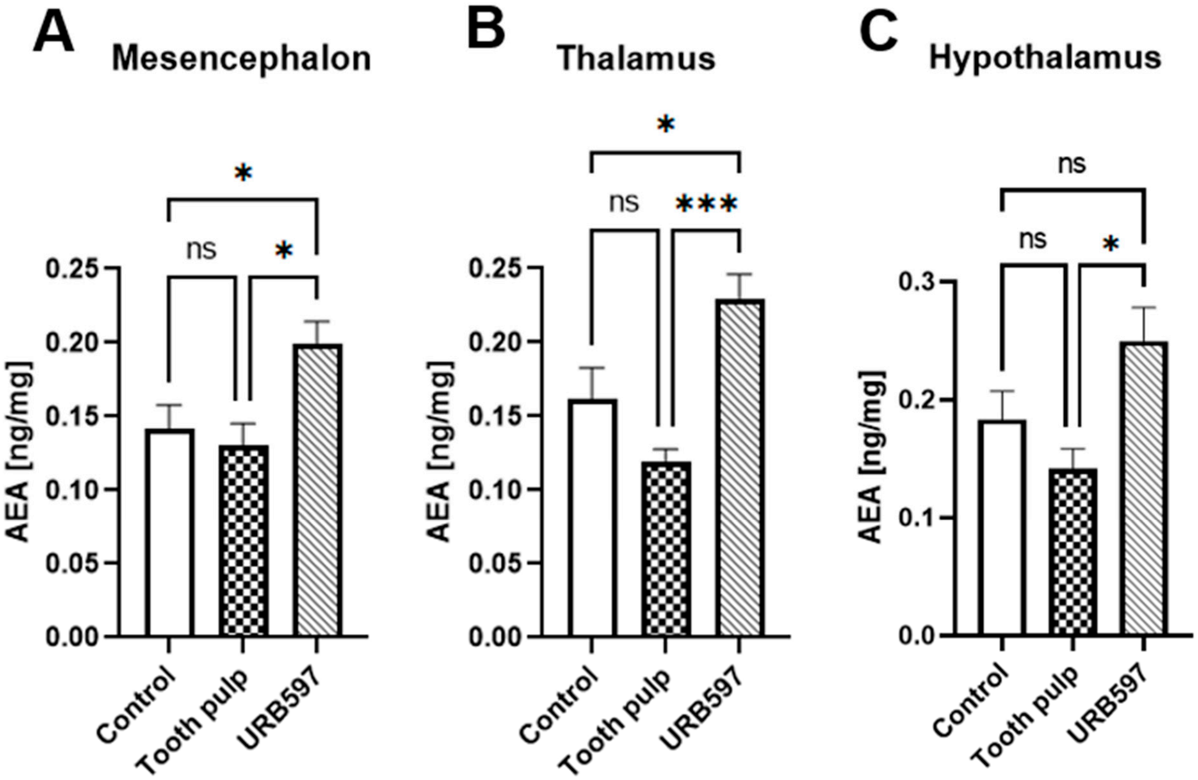

Supplementary Figure S2. AEA concentration normalized to milligram of total protein concentration in weighed tissue in controls and after tooth pulp and URB597 treatment. The data represent mean ± SEM of 6 rats per group. Statistical significance was assessed using one-way ANOVA and a post-hoc multiple comparison Tukey test. Asterisks denote significance level (* <0.05, **<0.01, *** <0.001, **** <0.0001, ns- not statistically significant).
